# Consecutive, Resting, Long-Duration Hyperoxic Exposures Alter Neuromuscular Responses During Maximal Strength Exercises in Trained Men

**DOI:** 10.3389/fphys.2019.00960

**Published:** 2019-07-31

**Authors:** Christopher M. Myers, Jeong-Su Kim, John P. Florian

**Affiliations:** ^1^Department of Nutrition, Food and Exercise Sciences, Florida State University, Tallahassee, FL, United States; ^2^Navy Experimental Diving Unit, Panama City Beach, FL, United States

**Keywords:** water immersion, neuromuscular performance, fatigue, electromyography, hyperoxia

## Abstract

**Purpose**: The main objective of this study was to investigate the physiological effects of repetitive diving-induced hyperoxic conditions at 1.35 atmospheres absolute (ATA) on neuromuscular strength performance. We hypothesized that following five days of consecutive, resting, long-duration (6 h or more) hyperoxic water immersions (WIs) neuromuscular strength performance would be reduced with a longer recovery time in comparison to previously reported normoxic WIs.

**Methods:** Thirteen (*n* = 13) active male divers [31.3 ± 1.7 (24–43) years, mean ± years] completed five consecutive days of 6-h resting WIs with 18-h surface intervals while breathing 100% O_2_ (*n* = 13) at 1.35 ATA. Skeletal muscle performance assessments occurred immediately before and after each WI and 24 and 72 h after the final WI. Performance assessments included maximum voluntary isometric contraction (MVIC) and maximal isokinetic (IK) knee extensions and elbow flexions, and isometric maximum handgrip (MHG) strength. Neuromuscular activation was also measured on the quadriceps, biceps brachii, and brachioradialis *via* surface electromyography (sEMG).

**Results:** MHG declined by 7.8% (*p* < 0.001) by WI 5 with performance returning to baseline by 24-h post-WI. Brachioradialis neuromuscular activation increased by 42% on WI 5. MVIC knee extension performance dropped by 4% (*p* = 0.001) on WI 3 with a 11% overall decrease in quadriceps neuromuscular activation. Maximal IK knee extension dropped by 3.3% on WI 5 with 9% drop in overall quadriceps activation during the same period. MVIC elbow flexion declined by 5.1% on WI 5 but returned to baseline by 72-h post-WI. Maximal IK elbow flexion performance dropped by 8.6% on WI 5 with a continual decline in biceps brachii neuromuscular activation of 24% on WI 5.

**Conclusion:** Consecutive, resting, long-duration hyperoxic WIs reduce muscular performance in multiple muscle groups and alter neuromuscular activation after 3 days of WI with performance adaptations recovering toward baseline by the end of the WI 5. However, neuromuscular activation remains decreased and appears to last beyond the 72-h post-WI recovery period.

## Introduction

Military, commercial, and technical divers are exposed to elevated hydrostatic pressures for extended periods, typically up to 6 h (Florian et al., 2016). The increased hydrostatic pressure and mechanical unloading during water immersion (WI) alter physiological function and may adversely impact physical performance after water egress (Myers et al., 2018). Furthermore, 100% oxygen may be used as the inspired gas to prevent nitrogen narcosis or mitigate the need for decompression (Brubakk et al., 2003). However, exposures to increased partial pressure of oxygen (hyperoxia) in addition to WI exacerbate changes to human performance.

Previously, we reported significant changes to neuromuscular maximum strength performance after 5 consecutive days of long-duration (6 h and greater) WIs (WI 1, 2, 3, 4, and 5) with subjects breathing air at 1.35 atmospheres absolute (ATA) (Myers et al., 2018). Maximal voluntary isometric contraction (MVIC) knee extension and elbow flexion torque decreased by 6 and 2%, respectively, by WI 3 of the dive week (Myers et al., 2018). Maximal isokinetic (IK) knee extension torque increased by 11 and 5% post-WI on WI 3 and WI 5 with greater neuromuscular activation post-WI than pre-WI. Maximum IK elbow flexion torque did not change; however, biceps brachii neuromuscular activation was greater post-WI than pre-WI (Myers et al., 2018). Maximum handgrip (MHG) strength force output was 4% greater post-WI than pre-WI with increased brachioradialis activation (Myers et al., 2018). The exposure to the microgravity environment caused noticeable increases in neuromuscular activation and decrements to maximal strength performance of load bearing muscles on WI 3 with full recovery occurring 3 days after the last WI. The non-load bearing muscles had increased neuromuscular activation but maintained performance. In both cases, the load-bearing and non-load-bearing muscle groups worked harder to maintain performance (Myers et al., 2018).

Florian et al. have demonstrated that consecutive, resting hyperoxic exposures to increased partial pressure of O_2_ (PO_2_) at 1.35 ATA, both dry in a hyperbaric chamber and immersed in water, cause significant reductions to human exercise performance (Florian et al., 2012, 2014). For example, 24 h after repeated exposure, time to exhaustion during 85% VO_2max_ treadmill testing decreased in the O_2_ group by 26% versus 5% in the air group (Florian, 2010), suggesting that these decrements are predominantly caused by hyperoxic exposure (Florian et al., 2012). Similar decrements to MHG strength were exhibited in the divers after resting, long-duration dry chamber, and WIs. The O_2_ WI dive group had an 8% reduction (O_2_ baseline 124.2 ± 3.5 lbs) in overall MHG strength average during 2–5 days (114.6 ± 1.7 lbs) during the dive week (Florian et al., 2011). Thus, reductions in MHG strength and cardiovascular endurance are exacerbated after repeated long-duration dives with divers breathing 100% O_2_ at 1.35 ATA (Florian et al., 2014).

Hyperoxic conditions also thought to change the resting potential of the motor neuron (Jammes et al., 2003; Brerro-Saby et al., 2010). Brerro-Saby et al. (2010) demonstrated reductions in electromyographic tonic vibratory response after breathing 100% O_2_ for 50 min in normobaric conditions, and Jammes et al. (2003) demonstrated 100% O_2_ exposures at 1.15 ATA in normal divers can cause decreases in conduction velocity time and increases in M-wave amplitude. In conjunction with these findings, Amann et al., 2006a demonstrated a significant reduction in myoelectric activation during fatiguing exercise with hyperoxic exposures when compared to normoxic and hypoxic exposures (Amann et al., 2006a,b; Amann and Calbet, 2008) and experienced changes in M-wave activity (Amann et al., 2006a). These studies have presented that hyperoxic exposures could cause significant changes to neuromuscular activation and possible changes to the motor neuron resting potential.

Although previous research into resting hyperoxic WIs has demonstrated decreases in aerobic performance and MHG strength, there is a paucity of information on the effects to broader neuromuscular performance. Therefore, the purpose of this study was to investigate the effects of consecutive, resting, long-duration hyperoxic WIs at 1.35 ATA on load and non-load bearing neuromuscular strength performance. We hypothesized that hyperoxic WIs at 1.35 ATA would cause greater decrements to neuromuscular strength performance and delay recovery times in comparison to previously reported WIs with divers breathing air at 1.35 ATA. The results of this study will address current gaps in the body of knowledge on how long-duration, hyperoxic WIs affect neuromuscular strength and recovery and investigate the differences, if any, to load and non-load bearing muscles.

## Materials and Methods

### Subjects

Thirteen active males, ${\dot{V}O}_{2\max}$ = 52.5 ± 5.0 ml/kg/min (mean ± SEM), with the average age of 31 ± 2 years and with an average of 7 ± 5 years (mean ± SD) of diving experience participated in this study as shown in Table 1. Before starting the study, each subject underwent a health screening that included a general physical, electrocardiogram, complete metabolic panel, lipid profile, and blood pressure measurements. Exclusion criteria included any known pulmonary, cardiovascular, or metabolic diseases, alcoholism, asthma, and nicotine use. Subjects were instructed not to consume any prescription, over-the-counter drugs, or supplements for the duration of the study unless authorized by our on-staff medical doctor trained in diving physiology who also reviewed all subjects’ medical histories.

**Table 1:** Subject characteristics.

Subject demographics	
*n*	13
Age (year)	31.3 ± 1.7
Height (cm)	177.7 ± 1.8
Weight (kg)	81.4 ± 2.8
Body mass index (kg/m^2^)	25.7 ± 1.7
${\dot{V}O}_{2\max}$(ml/kg/min)	52.5 ± 5.0

*Across subject (n = 13) mean ± SEM is shown*.

### Study Design

Each subject abstained from food and drink except water for 2 h prior to their laboratory visits and did not perform any fatiguing exercise 48 h prior to any scheduled testing. During all testing sessions, subjects wore running shorts and T-shirts. Subjects completed 5 consecutive days of 6-h resting WIs, referred to as the dive-week, with 18-h surface intervals with follow-up physiological testing occurring 24 h and 72 h after the fifth WI (Myers et al., 2018). Physiological testing during the dive week occurred immediately before and after each WI (Myers et al., 2018). All testing was performed in a climate-controlled laboratory (21.1–22.8°C). Each subject consumed a small, standardized breakfast consisting of 69% carbohydrate, 19% fat, and 12% protein (350 kcal) and 236 ml of orange juice after the pre-WI physiological testing (Myers et al., 2018). Before entering the test pool, each subject voided his bladder, weighed, and donned a condom catheter and urine bag to collect urine during the WI (Florian et al., 2012). Subjects breathed 100% O_2_ at 1.35 ATA while resting in a reclined position and did not perform any type of physical activity during the 6-h WI (Myers et al., 2018). After the WI, each subject removed the catheter, voided his bladder, dried off, and weighed (Faes et al., 2013; Florian et al., 2013; Myers et al., 2018). A medical doctor specializing in diving physiology was on site during each dive and evaluated each subject as needed after each WI for potential side effects. Post-WI physiological testing began immediately after weighing and evaluation by the medical doctor. No adverse effects were reported.

### Experimental Protocols

#### Familiarization

To minimize training effects during the intervention, all subjects visited the laboratory three times for familiarization sessions at least 72 h prior to the first WI. During the first two visits, each subject familiarized himself with the physiological testing protocols. Dynamometer configuration and sensor-placement measurements were also recorded during the first visit to ensure replication during subsequent visits (Myers et al., 2018).

#### Water Immersion

The WI protocol occurred as outlined in Florian et al. (2013). Subjects sat in reclining chairs in a thermoneutral (31.7–32.7°C) water tank (4.5 m) for 6 h. At the 3-h mark, subjects surfaced up to mid-chest for a 10-min break for a standardized lunch. The meal consisted of 64% carbohydrates, 24% fat, and 12% protein (475 kcal) and 500 ml of liquid (Florian et al., 2013; Myers et al., 2018). Once finished, each subject returned to the bottom of the tank to complete the WI (Florian, 2010; Myers et al., 2018).

#### Exercise Testing

Each subject underwent MVIC and maximal IK knee extension and elbow flexion and MHG assessment protocols. The Biodex System 4 Pro (Biodex Medical Systems, Shirley, NY, USA) was used for all knee extension and elbow flexion assessments. Subjects performed all exercise protocols on their right side, regardless of side dominance, and only concentric contractions were measured *via* the Biodex. The Biodex chair back angle was maintained at 85° for all performance testing (Harbo et al., 2012; Coggan et al., 2015; Myers et al., 2018). Subjects remained seated and strapped to the Biodex during all testing and recovery periods. Subjects did not grasp the Biodex chair or the straps nor were given any verbal encouragement during the exercise protocols.

#### Isometric MHG Protocol

Each subject performed the isometric MHG protocol while sitting on the Biodex prior to performing the knee extension and elbow flexion protocols. All MHG measurements were recorded using the Baseline BIMS^®^ HG dynamometer (White Plains, NY) and National Instruments NI USB 6210 module (Austin, TX). While conducting the exercise, the subject flexed his elbow at a 90° angle, wrist straight, and thumb orientated toward the ceiling while gripping the dynamometer. Each subject performed five consecutive 3-s MHGs with 30 s rest in between each repetition (Faes et al., 2013; Florian et al., 2016; Myers et al., 2018). The maximum force (kg) of each handgrip was recorded in National Instruments Labview (Austin, TX). Each subject rested for 3 min before beginning the MVIC knee extension.

#### Maximum Voluntary Isometric Contraction Knee Extension Protocol

The Biodex knee extension attachment was set at 70° (Denis et al., 2011; Coggan et al., 2015; Myers et al., 2018). Each subject performed one set of five consecutive 5-s MVIC contractions with 30 s rest between each repetition. The maximum torque (Nm) was recorded (Denis et al., 2011; Coggan et al., 2015; Myers et al., 2018). Upon completion, each subject rested for 3 min before performing the IK knee extension protocol.

#### Maximum Isokinetic Knee Extension Protocol

The subject’s position in the Biodex chair and attachment length did not differ from the MVIC positioning during the maximal IK knee extension assessment. Each subject performed two sets of five repetitions at a set angular rate of 60°/s concentric and 300°/s eccentric with 3 min of rest between each set (Findley et al., 2006; Powers and Jackson, 2008; Sahin et al., 2008; Denis et al., 2011; Harbo et al., 2012; Myers et al., 2018). The maximum torque (Nm) was recorded and analyzed (Denis et al., 2011; Coggan et al., 2015; Myers et al., 2018). Upon completion of the IK protocol, the subject rested for 3 min before continuing into the elbow flexion protocols.

#### Maximum Voluntary Isometric Contraction Elbow Flexion Protocol

With the elbow attachment set at 30°, each subject performed one set of five 5-s MVIC contractions with 30 s rest between each repetition (Drury et al., 2006; Myers et al., 2018). The maximum torque (Nm) was recorded and analyzed. Upon completion, each subject rested for 3 min before performing the subsequent protocol.

#### Maximum Isokinetic Elbow Flexion Protocol

After the 3-min recovery period, each subject performed two sets of five repetitions at a set angular rate of 60°/s concentric and 300°/s eccentric (Drury et al., 2006; Myers et al., 2018) with 3 min of rest between each set. The maximum torque (Nm) was recorded and analyzed.

#### Surface Electromyography

**Figure 1:**
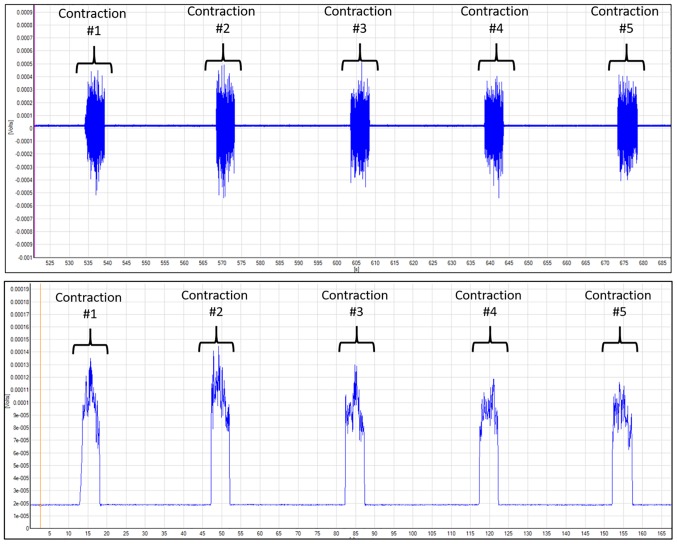
Depiction of vastus lateralis SEMG data for MVIC knee extension. The blue line is the electrical signal collected by the SEMG sensor during the exercise. Top figure depicts the raw SEMG data. Bottom figure shows the data that were transformed using the root mean squared (RMS) script within the Delsys Acknowledge software. The RMS method transforms the raw data to calculate the neuromuscular amplitude during the contraction.

Five sEMG (Delsys Trigno Wireless EMG systems, Boston, MA, USA) sensors were used to record neuromuscular activity during the exercise protocols (Myers et al., 2018). To ensure proper electrical conductance, the sEMG sites were shaved and cleaned with an alcohol pad. Sensors were attached the vastus lateralis, rectus femoris, vastus medialis, brachioradialis, and lateral head of the biceps brachii muscles using specialized, double-adhesive tape (Delsys Trigno adhesive, Boston, MA, USA). All sEMG signals were pre-amplified (×100), amplified (×2), band-pass filtered (10–1,000 Hz), and sampled at 2,000 Hz with EMG works software (version 4.1.7, Boston, MA, USA). As shown in Figure 1, raw sEMG data were converted using Fourier transformation root mean squared (RMS) script *via* EMG Works Analysis (Delsys, Boston, MA, USA). The raw sEMG amplitude data for all previously mentioned exercise protocols were normalized to baseline measurements. The isometric and isokinetic normalized amplitudes were reported in percent and used for statistical analysis.

#### Data Collection

All exercise testing occurred pre- and post-WI of 1, 3, and 5 and 24- and 72-h post-WI. Exercise testing was limited to these days to minimize testing induced fatigue on the subjects. Data collected during the pre-WI 1 session were used as the baseline measurements for comparison (Myers et al., 2018).

### Statistical Analysis

A 2 × 3 (pre/post-WI × day) repeated-measures analysis of variance (ANOVA) model was conducted to determine the effect of inspired gas on human performance after 5 consecutive days of WIs (Myers et al., 2018). For the dive week recovery period, a 1 × 3 repeated-measures ANOVA model was utilized to analyze recovery responses at 24- and 72-h post-WI (day main effect) compared to pre-WI 1 (baseline) (Myers et al., 2018). When appropriate, Bonferroni correction was used to identify the differences between main effects during post-hoc analysis. The SPSS Faculty Pack (version 23, IBM, Ontario, Canada) was used for all statistical analyses with the level of significance set at 0.05. Data are reported in means ± SEM.

## Results

### Subject Characteristics and Weight Loss

Subjects lost an average of 1.55 ± 1.4 kg during the overall testing period.

### Isometric MHG

#### Force Outputs

**Figure 2:**
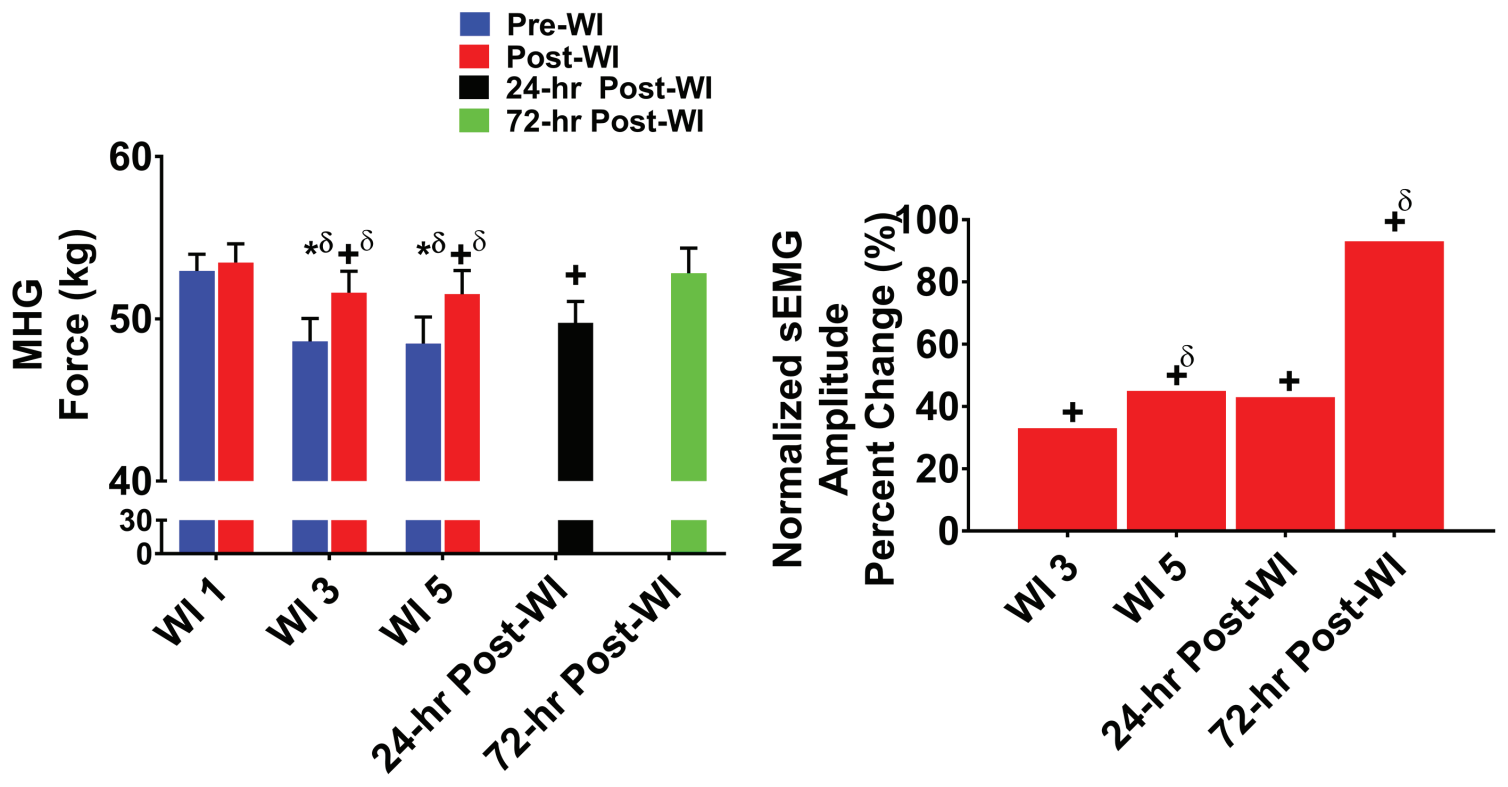
Maximum handgrip (MHG) results. Across subject (*n* = 13) mean ± SEM is shown. Left graph – performance (force): post-WI MHG performance was greater when compared to pre-WI. Additionally, performance declined on WI 3 and 5 when compared to WI 1. Performance for both groups returned to baseline performance by 72-h post-WI. “*” denotes pre/post-WI main effect (*p* < 0.05). “+” only denotes time main effect (*p* < 0.05). Main effect symbol with “δ” denotes (*p* < 0.001). Right graph – normalized sEMG day percent change: brachioradialis neuromuscular activation increased each day when compared to WI 1. “+” denotes time main effect (*p* < 0.05). Main effect symbol with “δ” denotes (*p* < 0.001).

Figure 2 illustrates the MHG force and brachioradialis sEMG results for the O_2_ groups across the testing period. Post-WI performance on WIs 3 and 5 was 4.1% greater (*p* < 0.001) than pre-WI performance for the same time points. Additionally, on WIs 3 and 5, there were 8.6% (*p* < 0.001) and 7.8% (*p* < 0.001) decreases in MHG, respectively. During the recovery period, a time main effect occurred where performance on 24-h post-WI was 6% lower (*p* < 0.016) than pre-WI 1 baseline (BL).

#### Neuromuscular Performance

In Figure 2, the brachioradialis normalized amplitude increased by +34% (*p* = 0.002) on WI 3 and by +42% (*p* < 0.001) on WI 5 when compared to WI 1. The 24- and 72-h post-WI sEMG amplitude was +49% (*p* = 0.001) and + 84% (*p* < 0.001) greater, respectively, when compared to BL.

### Maximum Voluntary Isometric Contraction Knee Extension

#### Torque Output

**Figure 3:**
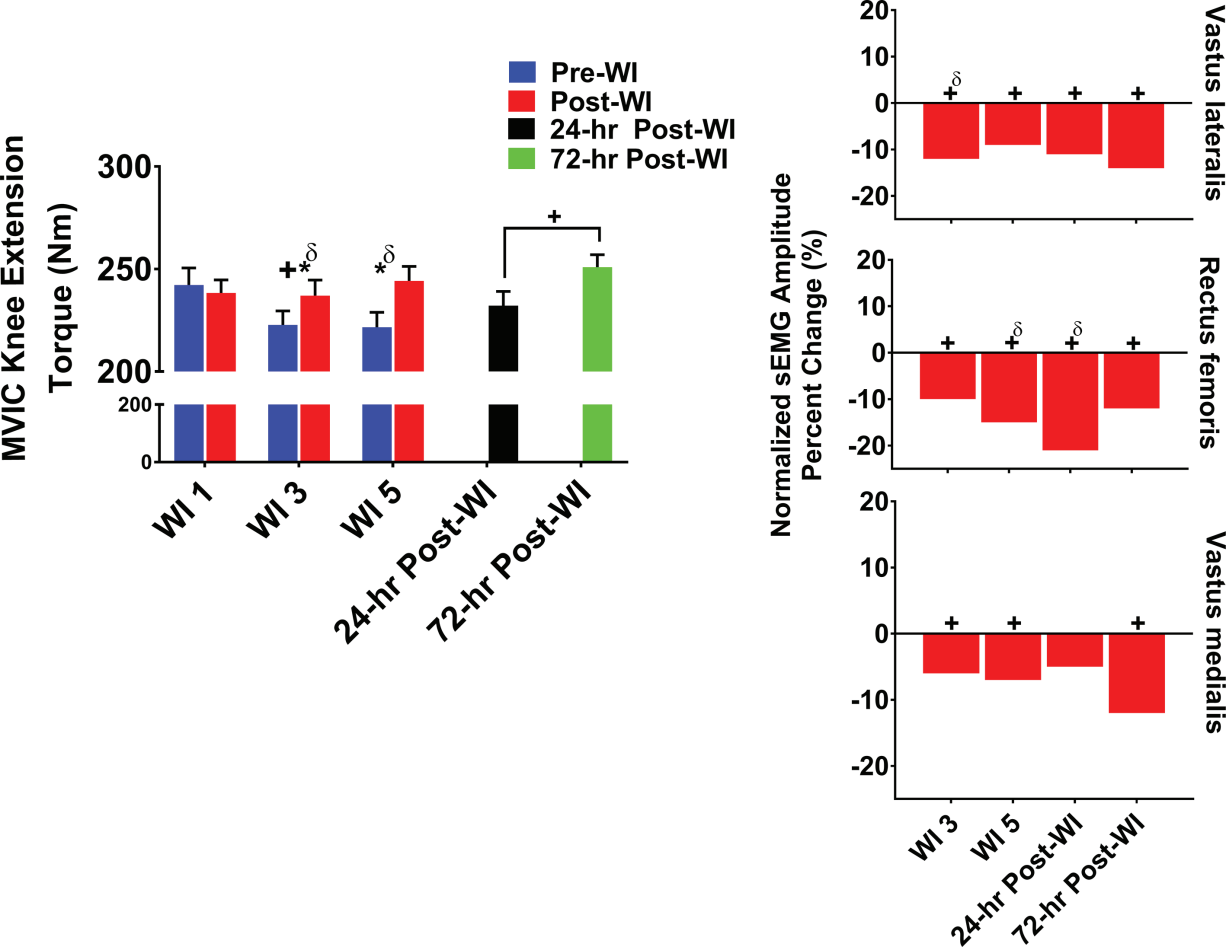
MVIC knee extension results. Across subject (*n* = 13) mean ± SEM is shown. Left graph – performance (force): post-WI performance was greater than pre-WI performance on WI 3 and 5. Additionally, performance declined by WI 3 but returned to baseline by WI 5. “*” denotes pre/post-WI main effect (*p* < 0.05). “+” only denotes time main effect (*p* < 0.05). Main effect symbol with “δ” denotes (*p* < 0.001). Right graph – normalized sEMG day percent change: neuromuscular activation decreased throughout the dive week and through the recovery period when compared to WI 1 for all three knee extensors. “+” denotes time main effect (*p* < 0.05). Main effect symbol with “δ” denotes (*p* < 0.001).

The MVIC knee extension torque and vastus lateralis, rectus femoris, and vastus medialis sEMG results are illustrated in Figure 3. A day main effect occurred on WI 3 where performance dropped −4% (*p* = 0.001) when compared to WI 1. Additionally, post-WI performance was 4% (*p* < 0.001) greater than pre-WI performance as shown in Figure 3. During the dive week recovery period, a time main effect (*p* = 0.012) was present with no difference between 24- and 72-h post-WI performances when compared to BL.

#### Neuromuscular Performance

As presented in Figure 3, vastus lateralis amplitude on WI 3 was 11.4% (*p* < 0.001) lower and on WI 5 was 9.2% (*p* = 0.005) lower when compared to WI 1. During the dive week recovery period, 24- and 72-h post-WI vastus lateralis amplitudes were 14% (*p* = 0.002, *p* = 0.006) lower when compared to BL, respectively.

The rectus femoris amplitude dropped by 10% (*p* = 0.002) on WI 3 and 15% (*p* < 0.001) on WI 5 when compared to WI 1. During the dive week recovery period, the 24- and 72-h post-WI amplitudes were 22% (*p* < 0.001) and 12% (*p* < 0.001) lower than BL, respectively.

On WIs 3 and 5, vastus medialis amplitude dropped by 6% (*p* = 0.013) and 7% (*p* = 0.012), respectively, when compared to WI 1. During the dive week recovery period, 72-h post-WI amplitude was 12% (*p* < 0.001) lower than BL as illustrated in Figure 3.

### Maximum Isokinetic Knee Extension

#### Force Output

**Figure 4:**
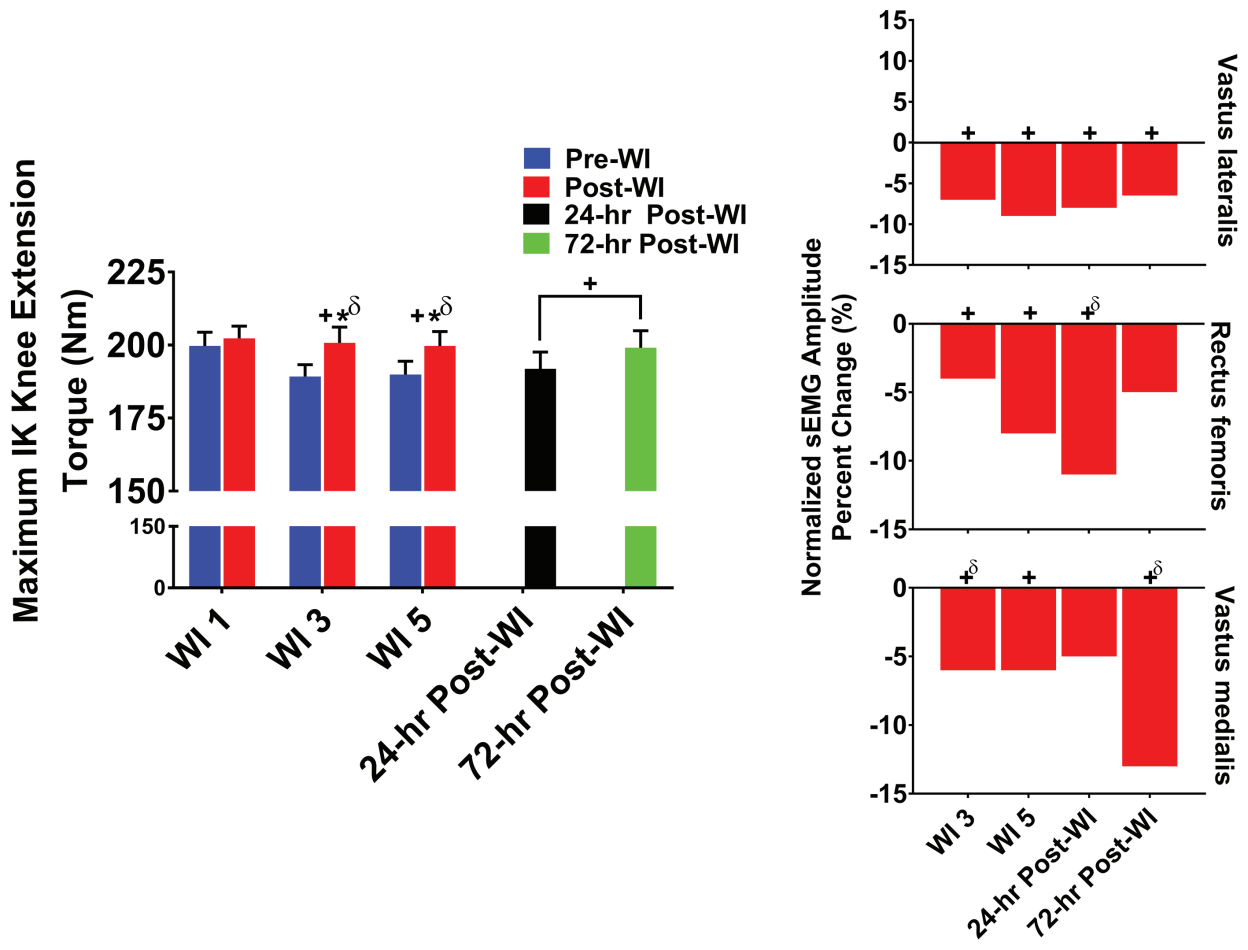
Maximum IK knee extension results. Across subject (*n* = 13) mean ± SEM is shown. Left graph – performance (torque): post-WI performance was greater than pre-WI on WIs 3 and 5. Overall performance declined on WIs 3 and 5 when compared to WI 1. “*” denotes pre/post-WI main effect (*p* < 0.05). “+” only denotes time main effect (*p* < 0.05). Main effect symbol with “δ” denotes (*p* < 0.001). Right graph – normalized sEMG day percent change: neuromuscular activation was significantly lower throughout the dive week and recovery period when compared to WI 1 for all three knee extensors. “+” denotes time main effect (*p* < 0.05). Main effect symbol with “δ” denotes (*p* < 0.001).

As shown in Figure 4, overall post-WI performance was 4% greater (*p* < 0.001) than pre-WI. A time main effect was observed on WIs 3 and 5 with a 3.3% (*p* = 0.008) drop in performance on both testing days. Additionally, a time main effect occurred during the recovery period (*p* = 0.032) with no difference found between BL versus 24- and 72-h post-WI.

#### Neuromuscular Performance

Vastus lateralis post-WI normalized amplitude was 8.6% (*p* < 0.001) greater than pre-WI as presented in Table 2. As illustrated in Figure 3, vastus lateralis amplitude on WIs 3 and 5 was 6.6% (*p* = 0.013) and 9.3% (*p* = 0.014) lower than WI 1, respectively. During the dive recovery period, 24-h post-WI was 8% (*p* < 0.001), and 72-h post-WI was 6.5% (*p* = 0.025) lower than BL.

**Table 2:** Direction of change for muscular performance variables and associated *p*. A ↑ or ↓ is shown for a *p* < 0.05, and ↑↑ or ↓↓ is shown for *p* < 0.001. The overall values show the differential effects of hyperoxic water immersions (WIs).

Exercise	Summary of results
**MHG**	
Performance (kg) – Day:	↓
NA (%) – Day:	↑↑
Performance (kg) – Pre-WI v Post-WI:	Post > Pre
**MVIC knee extension**	
Performance (Nm) – Day:	↔
NA (%) – Day:	VL: ↓
	RF: ↓↓
	VM: ↓
Performance (kg) – Pre-WI v Post-WI:	Post > Pre
**Maximal IK knee extension**	
Performance (Nm) – Day:	↓
NA (%) – Day:	VL: ↓
	RF: ↓↓
	VM: ↓↓
Performance (Nm) – Pre-WI v Post-WI:	Post > Pre
**MVIC elbow flexion**	
Performance (Nm) – Day:	↓↓
NA (%) – Day:	↓↓
Performance (Nm) – Pre-WI v Post-WI:	Post > Pre
**Maximal IK elbow flexion**	
Performance (Nm) – Day:	↓↓
NA (%) – Day:	↓↓
Performance (Nm) – Pre-WI v Post-WI:	Post > Pre

Rectus femoris presented with no pre-/post-WI main effect; however, a time main effect was present. On WI 3, rectus femoris amplitude was 4% (*p* = 0.010) lower than WI 1 as shown in Figure 4. Additionally, WI 5 amplitude was 8% (*p* = 0.004) lower than WI 1. During the recovery period, 24-h post-WI was 11% (*p* < 0.001) lower than BL.

The vastus medialis post-WI amplitude was 8.7% (*p* < 0.001) greater than pre-WI. WI 3 and 5 amplitudes were 6% (*p* < 0.001) and 6.1% (*p* = 0.009) lower than WI 1, respectively. During the dive week recovery period, the 72-h post-WI amplitude was 13% (*p* < 0.001) lower than BL.

### Maximum Voluntary Isometric Contraction Elbow Flexion

#### Force Outputs

**Figure 5:**
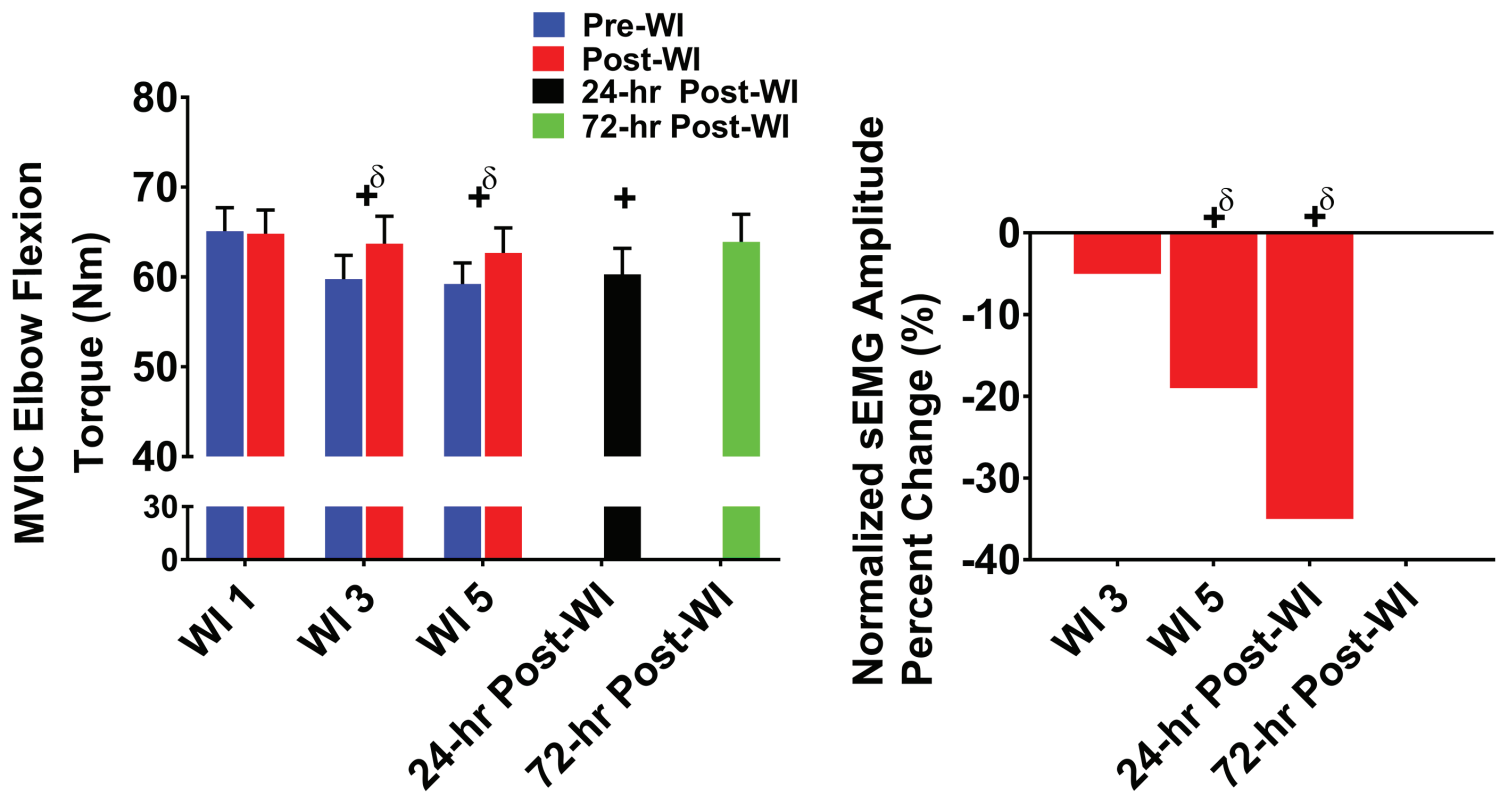
MVIC elbow flexion results. Across subject (*n* = 13) mean ± SEM is shown. Left graph – performance (force): overall performance declined throughout the entire dive week and did not recover until 72-h post-WI. “+” denotes time main effect (*p* < 0.05). Main effect symbol with “δ” denotes (*p* < 0.001). Right graph – Normalized sEMG day percent change: biceps brachii neuromuscular activation significantly declined throughout the dive week and on 24-h post-WI recovery period when compared to WI 1. “+” denotes time main effect (*p* < 0.05). Main effect symbol with “δ” denotes (*p* < 0.001).

In Figure 5, MVIC elbow flexion performance decreased by 5% (*p* < 0.001) on WI 3 and by 5.1% (*p* < 0.001) on WI 5 when compared to WI 1. During the dive week recovery period, 24-h post-WI performance was 7.2% (*p* = 0.001) lower than BL, but returned to BL by 72-h post-WI.

#### Neuromuscular Performance

The biceps brachii amplitude on WI 5 was 18.2% (*p* < 0.001) lower than WI 1. During the dive week recovery period, 24-h post-WI amplitude was 34.7% (*p* < 0.001) lower than BL.

### Maximum Isokinetic Elbow Flexion

#### Torque

**Figure 6:**
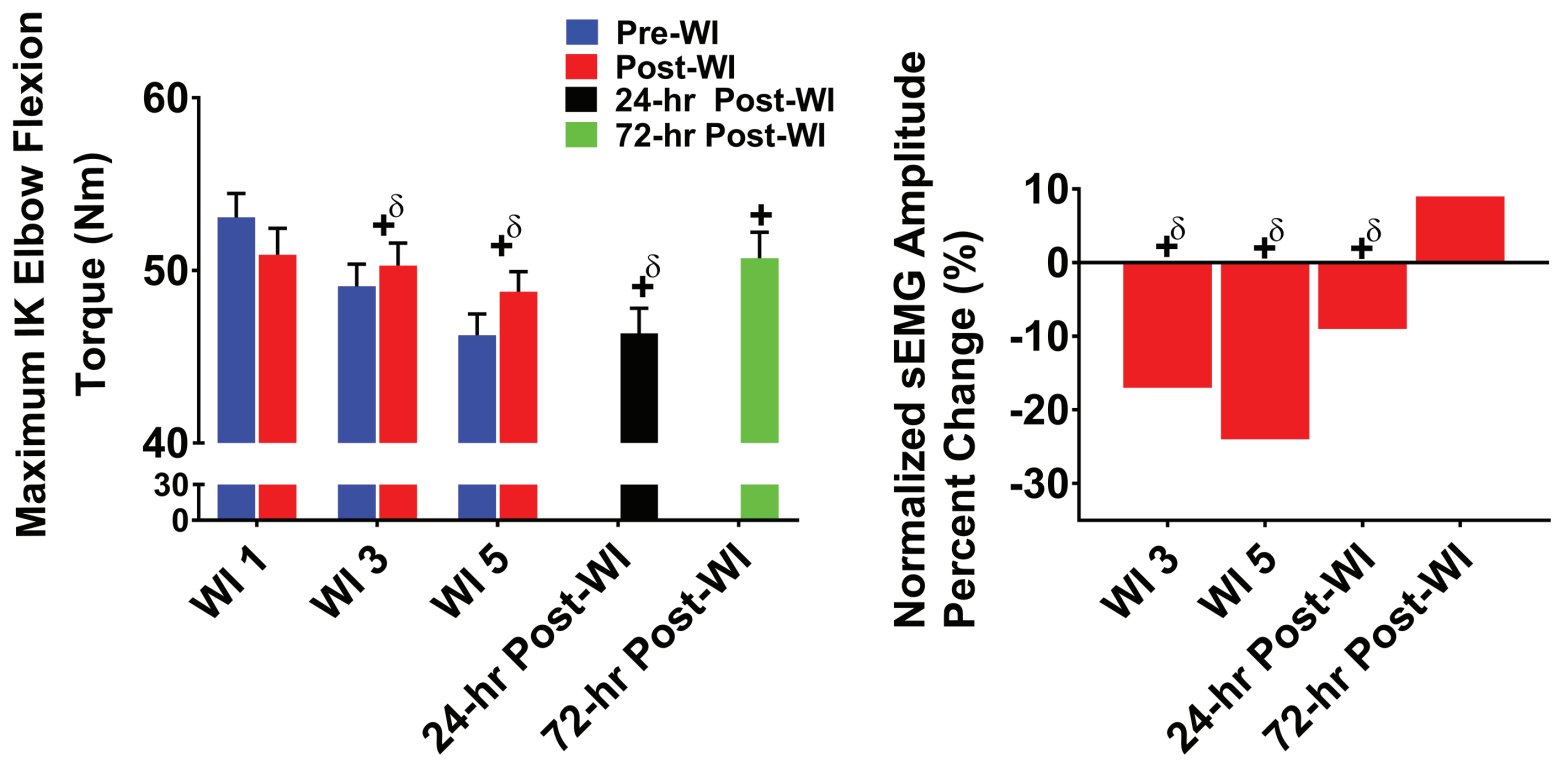
Maximum IK elbow flexion results. Across subject (*n* = 13) mean ± SEM is shown. Left graph – performance (torque): no group differences were present. Performance declined throughout the dive week and did not recover by 72-h post-WI. “+” denotes time main effect (*p* < 0.05). Main effect symbol with “δ” denotes (*p* < 0.001). Right graph – normalized sEMG day percent change: biceps brachii neuromuscular activation declined throughout the dive week and into 24-h post-WI when compared to WI 1. “+” denotes time main effect (*p* < 0.05). Main effect symbol with “δ” denotes (*p* < 0.001).

In Figure 6, maximal IK elbow flexion performance decreased by 4.4% (*p* < 0.001) on WI 3 and by 8.6% (*p* < 0.001) on WI 5 when compared to WI 1. Performance continued to be lower than BL by 12.5% (*p* < 0.001) at 24-h post-WI and 4.3% (*p* = 0.012) at 72-h post-WI.

#### Neuromuscular Performance

Biceps brachii post-WI normalized amplitude was 4% (*p* = 0.008) greater than pre-WI. Additionally, WI 3 and 5 were 17% (*p* < 0.001) and 24% (*p* < 0.001) lower than WI 1 as shown in Figure 6. At 24-h post-WI, the biceps brachii amplitude was 9% (*p* < 0.001) lower than BL.

## Discussion

The purpose of this study was to determine the extent to which consecutive, resting, long-duration 100% O_2_ WIs resulted in reduced muscular strength and increased recovery time in comparison to previously reported normoxic dives at 1.35 ATA. We hypothesized that neuromuscular strength performance would be reduced following repeated long-duration hyperoxic exposures with longer recovery time in comparison to normoxic exposures, thus illustrating a persistent reduction in performance. Our findings confirm the central hypothesis for this study. We found significant differences before and after the hyperoxic WIs, decreased performance throughout the dive week, and delayed recovery. Additionally, we unexpectedly observed significant decreases in neuromuscular activation in the large skeletal muscle groups.

Previously, we reported greater decrements to load-bearing muscle strength performance compared to non-load-bearing strength performance following repeated immersions with subjects breathing air at 1.35 ATA (Myers et al., 2018). This was not the case for this particular investigation. Non-load bearing muscles had greater decrements to maximal strength performance than load-bearing muscles. Overall, subject performance decreased by 4 to 8% in every strength exercise, and we observed similar decrements to strength performance beginning on WI 3. Normoxic WIs cause only 2% decrease in biceps performance on WI 3 with full recovery seen on WI 5 during the MVIC elbow flexion exercise; no changes were observed in the maximum IK elbow flexion and MHG exercises (Myers et al., 2018). In the current study, biceps performance dropped by 5 and 7% for the MVIC and maximum IK elbow flexion exercises, respectively. The MHG performance dropped by about 8% throughout the dive week after the hyperoxic exposures whereas performance did not change after the normoxic exposures (Myers et al., 2018). During normoxic WIs, knee extensor strength decreased by 6 and 3% on WIs 3 and 5 with full recovery observed on 72-h post-WI (Myers et al., 2018). No decrements were observed during maximum IK knee extension results. With this current study, we observed a 4% decrease on WI 3 in knee extensor strength performance during the MVIC knee extension exercise with recovery seen by WI 5. Performance decreased by 3% on WIs 3 and 5 for the maximal IK knee extension exercise as well. These day main effects indicate that hyperoxic WIs cause greater decrements than normoxic exposures. However, these results illustrate that the non-load bearing muscles are affected more extensively and require more recovery time than the load bearing muscles after hyperoxic WI exposures.

Another key difference between the normoxic and hyperoxic exposures is the effects on peripheral neuromuscular activation. In the case of the knee extensors and biceps brachii, neuromuscular activation decreased throughout the dive week even though performance declined. These responses are contrary to those seen after normoxic exposures where neuromuscular activation increased in the knee extensors, biceps brachii, and brachioradialis (Myers et al., 2018). Under normoxic conditions at 1.35 ATA, performance declined due to peripheral fatigue (Myers et al., 2018). However, with this study, we observed significant reductions in peripheral neuromuscular activation. The reductions in muscular strength performance in this study strongly suggest that they are caused by changes in the motor unit and how it activates the working muscle due the hyperoxic exposures at 1.35 ATA.

Similar changes to neuromuscular activation were seen in humans after dry normobaric and hyperbaric hyperoxic exposures (Jammes et al., 2003; Brerro-Saby et al., 2010). *In vitro* tests on invertebrate lobster skeletal muscle at 201.0 ATA have presented a 50% reduction in resting potential (Brubakk et al., 2003). However, at lower pressure, such as 10.9 ATA, only minimal decreases were reported with invertebrates (Brubakk et al., 2003). This decline to resting potential is characteristic of increased excitability, or hyperexcitability, of the skeletal muscle. Under hyperoxic conditions, neuromuscular hyperexcitability increases due to the change in resting potential (Jammes et al., 2003; Brerro-Saby et al., 2010). Jammes et al. demonstrated reduced peripheral nerve conduction velocity times with shortened M-wave duration and increased M-wave amplitude within the vastus lateralis in experienced recreational SCUBA divers after the subjects were exposed to 2 h of hyperoxic conditions at 1.15 ATA during resting dry dives (Jammes et al., 2003).

Brerro-Saby et al. took these results one-step further by exposing eight healthy human subjects to 100% O_2_ for 50 min in normobaric conditions. Before, during, and after the normobaric hyperoxic exposure, tonic vibratory response amplitude of the flexor digitorum superficialis was measured by sEMG. This study found after the short hyperoxic exposure, peripheral neuromuscular activation of the flexor digitorum superficialis was reduced after 30 and 50 min of exposure and continued to be depressed after 10 min of recovery (Brerro-Saby et al., 2010). Our study found similar decreases in neuromuscular activation in large muscles, but not in small muscles (i.e., brachioradialis, Figure 2B). The changes that we observed in the knee extensors were similar to what was reported by Amann et al. (2006a,b). These results demonstrate how hyperoxia affects peripheral nervous system signaling. While not being able to account for the change in neuromuscular activation for the brachioradialis, overall results generally show that hyperoxic exposures, not WI, cause these changes to peripheral neuromuscular activation.

Several key differences between the Jammes et al. and Brerro-Saby et al. studies and our study are the use of tonic vibration response and nerve conduction velocity methodologies, which we did not use in our study. Foremost, our findings and interpretations are based on our assessment of voluntary neuromuscular activation. The Jammes et al. and Brerro-Saby et al. studies used tonic vibration response for neuromuscular activation assessment and nerve conduction velocity. The use of nerve conduction velocity represents slightly different physiological variables than voluntary neuromuscular activation. Nerve conduction velocity evokes H and M-waves. The H-wave (Hoffman’s reflex) is the electrically evoked equivalent of the monosynaptic stretch reflex. The M-wave, also referred to as compound muscle action potential, is the direct motor response, which is routinely measured by electromyography (Leis and Schenk, 2013). In practice, the sEMG amplitudes measured in the Brerro-Saby et al. and our studies are measuring the M-wave. However, this is not directly the case with the Brerro-Saby et al. study. Tonic vibratory response reflects peripheral neuromuscular activation *via* vibration of the muscle spindle of the target muscle during sustained contraction. This method is an indirect method of measuring neuromuscular activation. We recorded neuromuscular activation directly *via* the electrical signaling properties of the motor unit. In both cases, the same result occurred. The increased amplitude and decreased duration of the M-wave (membrane excitability) along with decreased nerve conduction velocity (signal propagation) illustrate the changes to the peripheral nervous system after hyperoxic exposures (Jammes et al., 2003; Brerro-Saby et al., 2010). The changes seen in the M-wave suggest that the increased membrane resting potential (Brerro-Saby et al., 2010) signifies less impulse was needed to activate the muscular twitch. The reduced number of stimuli needed to cause muscular twitch could explain the decrease in sEMG amplitude seen in the Brerro-Saby et al. and our study.

The causality for the hyperoxic change in neuromuscular activation is unknown. What is known is that the sEMG signal measures the neuromuscular activation of the entire motor unit beneath the sensor to include the activation of the α- and γ-motor neurons and the muscle spindle from the peripheral nervous system (MacIntosh et al., 2006; Brerro-Saby et al., 2010; Merletti and Farina, 2016). Brerro-Saby et al. and Jammes et al. suggest that the interaction of free radicals, particularly, reactive oxygen species (ROS) interact with the α- and γ-motor neurons to cause a change in the resting membrane potential (Jammes et al., 2003; Brerro-Saby et al., 2010). Hyperoxic exposures are known to increase ROS and oxidative stress levels within the human body (Brubakk et al., 2003; Ooij et al., 2013), while hyperbaric hyperoxic conditions exacerbate the response further (Brubakk et al., 2003). Yet, the effects of hyperoxia appear to affect only the excitable structures within the peripheral nervous system and the skeletal muscle motor unit (Brubakk et al., 2003; Jammes et al., 2003; Brerro-Saby et al., 2010). This hypothesis is strengthened by results from Delliaux et al., 2009, demonstrating in animals that ROS donors do not affect the muscle spindle (Delliaux et al., 2009). Through the process of elimination, Brerro-Saby et al. and Jammes et al. state the α- and γ-motor neurons are the only excitable structures affected. Further research is necessary to examine the root cause of reduced neuromuscular activation due to hyperoxic exposures.

In every exercise, average performance values are greater post-dive than pre-dive. At first glance, the increased post-dive performance values appear to illustrate recovery after the hyperoxic exposures. However, this is not the case. In Figures 2–4, pre-dives are lower than post-dive. A similar characteristic is evident with Figures 5, 6, albeit the differences were not significant. Furthermore, in every case, the pre-dive values for WI 3 and 5 were lower than baseline. Rarely did the post-dive performance values surpass the baseline performance values. This shows how fatigue settles in after each dive, and performance is worsened the following day. Similarly, we reported previously post-dive increases of 4% for MHG and 11% for the maximum IK knee extension during normoxic water immersions (Myers et al., 2018). For this study, we observed a 4.1% post-dive increase for MHG, but only a 4% post-dive increase for the maximum IK knee extension (Figure 3). Further evidence of this is seen in the MHG and MVIC knee extension results (Figures 2, 3). This evidence strongly suggests a significant hyperoxic effect causing a delayed onset of fatigue. The MHG results from the Florian et al. had similar results (Florian et al., 2011). Florian et al. first suggested the delayed onset of fatigue as the causality for these pre-/post-dive performance differences after several hyperoxic WI exposures (Florian et al., 2011).

The mechanism for this decrement in pre-/post-dive performance is unknown. The data collected during this study cannot explain the differences in the pre-/post-dives results. One possibility may be caused by free radical generation caused by the hyperoxic exposures as established by the previously mentioned research (Brubakk et al., 2003; Jammes et al., 2003; Delliaux et al., 2009; Brerro-Saby et al., 2010). High levels of free radical generation are known to cause contractile dysfunction within skeletal muscle (Steinbacher and Eckl, 2015). Free radical oxygen species are known to affect calcium sensitivity of Troponin I in skeletal muscle (Steinbacher and Eckl, 2015). Reduced calcium sensitivity will reduce muscular contraction and force production. Furthermore, increased concentrations of ROS are known to cause changes to the S1 fragment of the myosin head (Steinbacher and Eckl, 2015). Changes to this particular protein block the attachment of ATP (Steinbacher and Eckl, 2015). Without ATP, the myosin head will not release from the actin filament during the cross-bridge cycle (Powers and Jackson, 2008). Further investigation is necessary to understand the underlying causes of the pre-/post-dive performance differences.

## Conclusion

The present study demonstrates that the effects of resting, consecutive, long-duration hyperoxic immersions have on neuromuscular performance. The results of this study confirm that resting hyperoxic exposures cause significant decrements to the skeletal muscle strength performance and delays recovery. Furthermore, these types of hyperoxic exposures cause significant decreases to neuromuscular activation amplitude with large muscle groups. This amplitude change can be explained through hyperexcitability. More electrophysiological research is necessary to fully examine the underlying mechanisms involved with this change.

## Ethics Statement

Approval for the study was given by the Institutional Review Boards for the Navy Experimental Diving Unit and Florida State University. Prior to starting the study, each subject provided a written informed consent, and all procedures conformed to the Declaration of Helsinki.

## Author Contributions

CM, J-SK, and JF conceived and designed the research study. All authors conducted the research. CM analyzed the data and wrote the manuscript. All authors read and approved the manuscript.

### Conflict of Interest Statement

The authors declare that the research was conducted in the absence of any commercial or financial relationships that could be construed as a potential conflict of interest.
